# Association of Encephalitis With Viruses in Cerebrospinal Fluid in Children: A Case Series

**DOI:** 10.7759/cureus.83891

**Published:** 2025-05-11

**Authors:** Kenta Kuruma, Hanako Funakoshi, Meiwa Shibata, Kazue Kinoshita, Yuho Horikoshi

**Affiliations:** 1 Division of Infectious Diseases, Tokyo Metropolitan Children’s Medical Center, Tokyo, JPN; 2 Department of Pediatrics, Division of Infectious Diseases, Tokyo Metropolitan Children’s Medical Center, Tokyo, JPN; 3 Division of Molecular Laboratory, Tokyo Metropolitan Children’s Medical Center, Tokyo, JPN

**Keywords:** acute encephalitis, csf infection, real-time pcr, virus, virus research

## Abstract

Introduction

The application of polymerase chain reaction (PCR) to cerebrospinal fluid (CSF) analysis has enabled the direct detection of neuropathogenic viruses in patients with meningitis or encephalitis. However, the presence of a virus in the CSF does not necessarily confirm a diagnosis of encephalitis. Clinical judgment is required to determine whether neurological follow-up is warranted. This study aimed to investigate the incidence of encephalitis among patients with viral detection in CSF and to evaluate the relationship between viral load and disease occurrence.

Methods

Patients under 16 years of age with a virus detected in CSF by PCR between April 2012 and March 2023 at the Tokyo Metropolitan Children’s Medical Center were included in this study. Lumbar puncture and viral PCR testing were performed in patients with a clinical suspicion of meningitis or encephalitis. Encephalitis was diagnosed according to the criteria established by the International Encephalitis Consortium. Data on patient demographics, PCR results, viral load, and final diagnosis were extracted from electronic medical records. Viral species and viral loads were compared between patients diagnosed with and without encephalitis.

Results

A total of 64 patients had a virus detected in CSF between April 2012 and March 2023. Of these, 32 patients (50%) were diagnosed with encephalitis. The median age of patients with encephalitis was 13.5 months (interquartile range {IQR}: 1.75-33 months), compared to nine months (IQR: 0-22.5 months) in those without encephalitis. Among patients with encephalitis, the most frequently detected viruses were human herpesvirus 6 (HHV-6) in 13 patients, enterovirus (EV) in eight, and human parechovirus (HPeV) in one. In patients without encephalitis, HHV-6 was detected in eight cases, enterovirus in four, and HPeV in 13. Patients with encephalitis due to HHV-6 showed a higher viral load (median: 4,800 copies/mL) compared to those without encephalitis (median: 1,070 copies/mL; p = 0.019).

Conclusion

Encephalitis was diagnosed in 50% of patients with viral detection in CSF. HHV-6 and enterovirus were the most commonly identified pathogens. Elevated HHV-6 viral load may be associated with the presence of encephalitis.

## Introduction

Since the development of polymerase chain reaction (PCR) in 1983, advancements in molecular techniques, particularly automated multiplex PCR, have enabled the rapid identification of multiple pathogens in pediatric central nervous system (CNS) infections [1-3]. However, the broad range of targets included in multiplex PCR panels may result in the detection of microorganisms that are not causative agents of CNS disease [4].

Febrile seizures are common among children in Japan and other parts of East Asia and globally [5]. In some cases, complex febrile seizures, especially those progressing to status epilepticus, can clinically resemble encephalitis. PCR is highly sensitive and capable of detecting minute quantities of viral genetic material, including viruses that cause viremia in the cerebrospinal fluid (CSF) [6,7]. One study reported that multiplex PCR was performed in children with febrile convulsions and found no clinical differences between those with and without viral detection [8]. Additionally, positive PCR results for enteroviruses (EV) have been observed in young infants even when CSF cell counts were within normal limits [9].

Therefore, clinical interpretation should incorporate epidemiological context, patient history, and additional laboratory findings to determine whether a detected microorganism is indeed the causative pathogen [10].

Currently, it remains unclear what proportion of patients with viral detection in CSF actually develop encephalitis. While some studies have reported an association between pneumonia and higher viral loads in respiratory infections, it is uncertain whether a similar relationship exists between viral load and encephalitis [11]. This study aimed to evaluate the frequency of encephalitis among patients with viral detection in CSF by real-time PCR and to assess whether viral load correlates with the occurrence of encephalitis.

## Materials and methods

Study protocol

A lumbar puncture was performed at the discretion of the attending clinician in patients admitted with fever, seizures, impaired consciousness, or shock suggestive of encephalitis or meningitis. In cases where a CNS infection was suspected, real-time PCR testing was conducted for the following viruses: herpes simplex virus 1/2 (HSV-1/2), human parechovirus (HPeV), enterovirus (EV), human herpesvirus 6 (HHV-6), human herpesvirus 7 (HHV-7), adenovirus (ADV), cytomegalovirus (CMV), Epstein-Barr virus (EBV), varicella zoster virus (VZV), and human parvovirus B19.

Encephalitis was diagnosed according to the criteria established by the International Encephalitis Consortium [12]. Meningitis was defined as the presence of clinical symptoms consistent with meningitis and a CSF leukocyte count exceeding 4×10⁶ cells/L [13].

This study included patients in whom viral presence was detected in CSF between April 2012 and March 2023 at the Tokyo Metropolitan Children’s Medical Center. Exclusion criteria were as follows: patients aged 16 years or older, CSF samples obtained at a referring hospital, insufficient CSF volume for real-time PCR testing, and the detection of bacteria or fungi in CSF culture. Cases in which CSF was contaminated with blood were also excluded. The Institutional Review Board (IRB) of Tokyo Metropolitan Children’s Medical Center issued approval 2023b-59.

Demographic and clinical data, including patient background, presenting symptoms, laboratory findings, real-time PCR results, and clinical outcomes, were extracted from electronic medical records. The primary outcome was the proportion of encephalitis cases among patients with viral detection in CSF. Secondary outcomes included comparisons of patient characteristics, types of viruses detected, and viral loads between patients with and without encephalitis.

Real-time PCR assay

Viral RNA and DNA were extracted from CSF samples using the QIAamp MinElute Virus Spin Kit (Qiagen, Valencia, CA). The quantification of viral load was performed through a real-time PCR assay utilizing the TaqMan probe method. A 20 µL reaction system was prepared using PrimeScript RT Master Mix (TaKaRa, San Jose, CA) and primer-probe mixes specific for HPeV, HHV-6/7, HSV-1/2, EV, human parvovirus B19, EBV, CMV, and ADV, provided by SayMed Inc. (Delhi, India). Viral RNA was quantified using real-time PCR based on a standard curve generated from serial dilutions of plasmids containing known copy numbers.

Statistical analyses

All statistical analyses were performed using the SPSS software (IBM Corp., Armonk, NY). Categorical variables were compared using the χ² test or Fisher’s exact test, as appropriate. A p-value of <0.05 was considered statistically significant. Data presented in the tables are primarily expressed as medians with corresponding first and third quartile values.

## Results

Primary outcome

**Figure 1:**
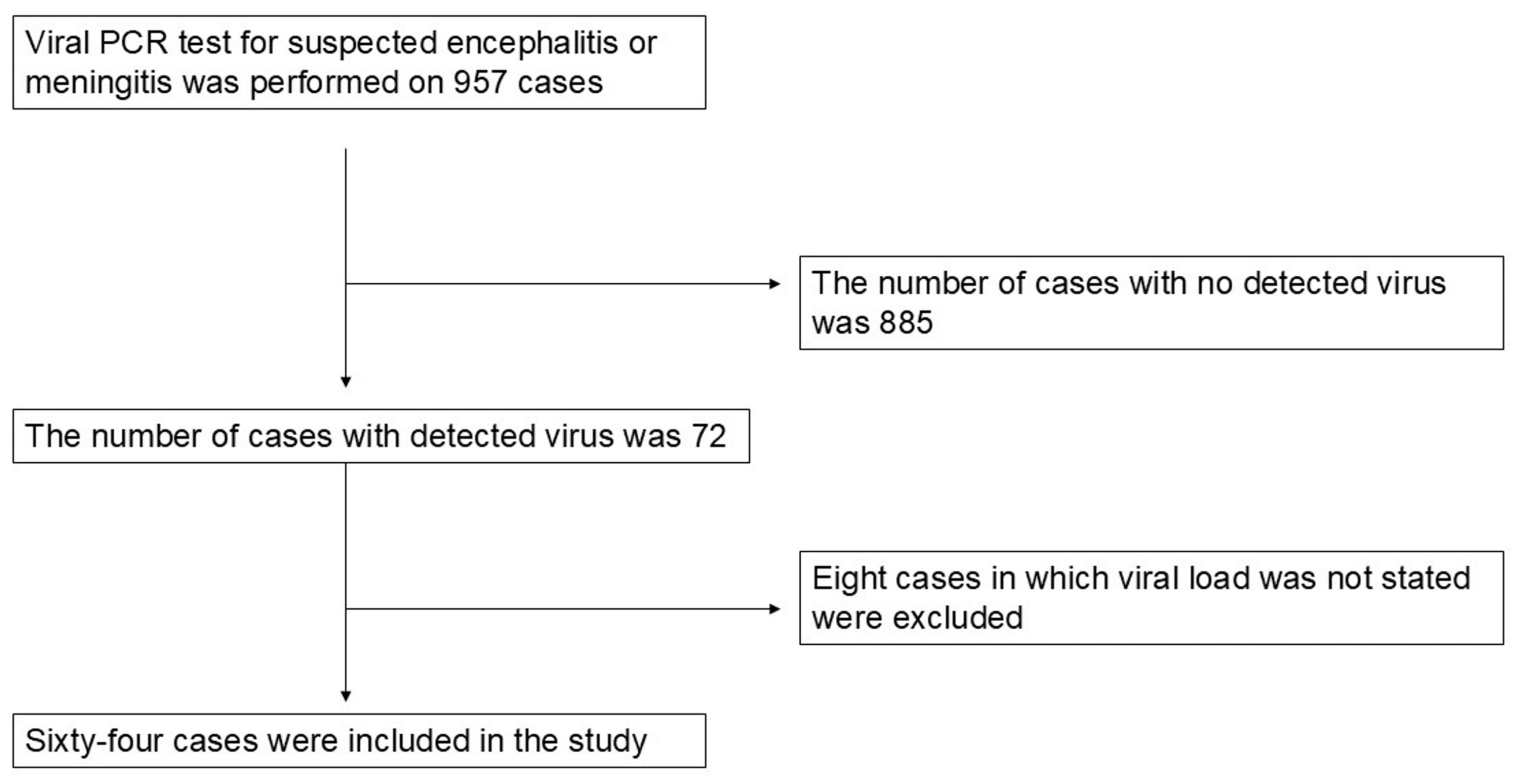
Study flowchart The figure presents the patient selection process in a flowchart, illustrating the inclusion and exclusion criteria applied in the study PCR: polymerase chain reaction

During the study period from April 2012 to March 2023, viral PCR testing of CSF was performed in 957 pediatric patients. Among these, 72 patients tested positive for at least one virus. After excluding eight patients due to incomplete viral load data, a total of 64 patients were included in the final analysis. Of these, 32 patients (50%) were diagnosed with encephalitis based on the criteria established by the International Encephalitis Consortium. No cases of meningitis were identified. Figure 1 illustrates the patient selection process in a flowchart, detailing the inclusion and exclusion criteria used in the study. Table 1 presents the final clinical diagnoses of patients with viral detection in CSF, showing that, in addition to encephalitis, common alternative diagnoses included sepsis (n = 18) and febrile seizures (n = 11).

**Table 1:** Final diagnosis and number of patients with a virus detected in CSF CSF: cerebrospinal fluid

Final diagnosis	Number
Encephalitis	32
Sepsis	18
Febrile seizure	11
Others	3

Secondary outcomes

Table 2 compares the demographic and clinical characteristics of patients with and without encephalitis. No statistically significant differences were observed between the two groups in terms of age, sex, or underlying medical conditions, such as immunodeficiency or prematurity. Notably, the viral load was significantly higher in the non-encephalitis group (median: 4,100 copies/mL) compared to the encephalitis group (median: 990 copies/mL), with a p-value of <0.01.

**Table 2:** Characteristics of patients with and without encephalitis IQR: interquartile range

	With encephalitis (N = 32)	Without encephalitis (N = 32)	P-value
Age (months) (IQR)	13.5 (1.75-33)	9 (0-22.5)	0.41
Percentage of female sex	18 (50%)	18 (50%)	1
Neurological disorder	0	1	0.32
Premature birth (<37 weeks）	5	2	0.28
Immunodeficiency	2	0	0.16
Viral load (copies/mL) (IQR)	990 (171-9,975)	4,100 (552-2,5000)	<0.01

The distribution of detected viruses is detailed in Table 3. HHV-6 was the most frequently detected virus in both groups, followed by EV and HPeV. Importantly, HPeV was significantly more prevalent among patients without encephalitis (p = 0.001).

**Table 3:** Types of virus detected in the groups with and without encephalitis HHV-6, human herpesvirus 6; HHV-7, human herpesvirus 7; VZV, varicella zoster virus; ADV, adenovirus; CMV, cytomegalovirus; HPeV, human parechovirus; EBV, Epstein-Barr virus; EV, enterovirus; HSV-1/2, herpes simplex virus 1/2

	With encephalitis (N = 32)	Without encephalitis (n = 32)	P-value
HHV-6	13	8	0.3
HHV-7	3	1	0.3
HSV-1/2	3	0	0.09
HPeV	1	15	0.001
EV	8	4	0.28
ADV	1	0	0.32
EBV	0	2	0.16
CMV	0	0	-
VZV	1	1	1
Human parvovirus B19	2	1	0.57

**Figure 2:**
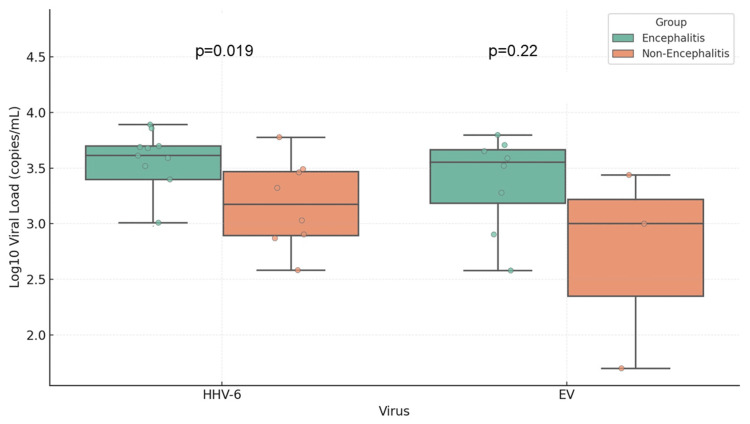
Comparison of viral load in cerebrospinal fluid by virus and encephalitis diagnosis The figure shows the comparison of viral load (log-transformed) between the encephalitis and non-encephalitis groups for human herpesvirus 6 (HHV-6) and enterovirus (EV). HHV-6 shows a significantly higher viral load in encephalitis cases (p = 0.019). EV does not show a significant difference (p = 0.22)

Figure 2 highlights the viral loads of HHV-6 and EV, the two most frequently detected viruses in the study. Among patients with HHV-6, those diagnosed with encephalitis had a significantly higher viral load (median: 4,800 copies/mL) compared to those without encephalitis (median: 1,070 copies/mL; p = 0.019). In contrast, no significant difference in EV viral load was observed between the encephalitis and non-encephalitis groups (p = 0.22).

## Discussion

In this study, half of the viruses detected in CSF did not cause encephalitis. In virus-specific analyses, only HPeV was significantly more common among patients without encephalitis. Previous studies have reported that laboratory findings in patients with HPeV in CSF often do not indicate abnormalities suggestive of CNS infection. It is possible that HPeV influenced the study outcomes due to its known ability to cause systemic infections and sepsis-like symptoms [14]. However, even after excluding HPeV cases, 35% of patients with viral detection in CSF did not present with CNS infection. Therefore, the presence of a virus in CSF alone does not necessarily imply causation of encephalitis.

Regardless of encephalitis status, HHV-6 was the most frequently detected virus, followed by HPeV and EV. A previous study reported that 30% of encephalitis cases were of infectious origin, with HSV being the most commonly identified pathogen [15]. This discrepancy may be due to regional differences in viral epidemiology. For example, a Japanese study reported that HHV-6 and the influenza virus were the most frequently detected viruses in patients with encephalitis [16]. Prior research has highlighted the difficulty in detecting the influenza virus in CSF, possibly due to the cytokine-mediated nature of influenza-associated encephalitis rather than direct viral invasion [17,18]. In line with this, influenza virus testing in CSF was not performed in the present study. Nevertheless, HHV-6 appears to be a major virus detected in CSF in pediatric patients in Japan.

Unexpectedly, viral loads in CSF were higher among patients without encephalitis. When analyzed by virus type, only VZV and HHV-7 showed a trend toward higher viral loads in the non-encephalitis group. However, the number of patients in these groups was small, limiting the clinical significance of this finding. As reported in previous studies, HPeV can be detected in CSF even in the absence of encephalitis [19]. The present study suggests that HHV-7 and VZV may exhibit similar characteristics.

The analysis of virus-specific viral loads revealed a statistically significant association between HHV-6 load and encephalitis. Previous studies have established HHV-6 as a causative agent of encephalitis, and its detection in CSF may reflect active neuropathogenic processes [20,21]. Some reports have also suggested a correlation between viral load and encephalitis in VZV and HSV infections [22,23]. In a similar manner, high HHV-6 viral load in CSF may be linked to encephalitis pathogenesis.

Conversely, no association was found between enteroviral load and encephalitis. A study of adult patients with EV-related CNS infections reported consistent inflammatory changes in CSF, such as elevated white cell counts and the disruption of the blood-CSF barrier [24]. As with influenza-related encephalitis, elevated CSF cytokine levels have been reported in EV-related encephalitis cases [25]. These findings suggest that enteroviral encephalopathy may be mediated more by cytokine activity than by direct viral effects, which may explain the lack of correlation between viral load and encephalitis in EV cases. However, this observation may also be influenced by the small sample size, highlighting the need for further research.

This study has several limitations. First, its retrospective design may have introduced selection bias regarding viral prevalence. However, the extended 11-year study period likely mitigated seasonal or epidemic-related fluctuations. Second, viral infections caused by pathogens not included in the PCR panel may have been missed. Nevertheless, the panel covered viruses commonly reported in Japan [16]. Third, the absence of cytokine measurements prevented the assessment of encephalitis driven by immune-mediated mechanisms, as seen in influenza and SARS-CoV-2 cases [26,27]. Finally, the study was conducted at a single institution, limiting the generalizability of the findings due to potential regional variations in prevalent viruses.

## Conclusions

Of the cases in which the virus was identified in CSF, encephalitis was diagnosed in half of the cases. Despite the detection of viruses in CSF, the instances of concomitant encephalitis are few. HHV-6 and enterovirus were the two most commonly detected viruses. The non-encephalitis group demonstrated a significantly higher viral load than the encephalitis group, but the patients with encephalitis with HHV-6 had a significantly higher viral load. The viruses identified in the CSF of the present cohort need to be evaluated individually.
